# Development and acceptability testing of ready-to-use supplementary food made from locally available food ingredients in Bangladesh

**DOI:** 10.1186/1471-2431-14-164

**Published:** 2014-06-27

**Authors:** Tahmeed Ahmed, Nuzhat Choudhury, M Iqbal Hossain, Nattapol Tangsuphoom, M Munirul Islam, Saskia de Pee, Georg Steiger, Rachel Fuli, Shafiqul A M Sarker, Monira Parveen, Keith P West, Parul Christian

**Affiliations:** 1 Centre for Nutrition and Food Security, icddr,b, 68 Shaheed Tajuddin Ahmed Sarani, Mohakhali, Dhaka 1212, Bangladesh; 2 James P. Grant School of Public Health, BRAC University, Dhaka, Bangladesh; 3 Food Science Unit, Institute of Nutrition, Mahidol University, Nakhon Pathom 73170, Thailand; 4 World Food Programme, Via Cesare Giulio Viola 68/70, Rome 00148, Italy; 5 DSM Nutritional Products Ltd, 4303 Kaiseraugst, Switzerland; 6 World Food Programme, E/8A Rokeya Sharani, Agargaon, Sher-e-Bangla Nagar Dhaka-1207, Bangladesh; 7 Center for Human Nutrition, Department of International Health, Johns Hopkins Bloomberg School of Public Health, Baltimore, MD 21205, USA

**Keywords:** Ready-to-use supplementary food (RUSF), Local food ingredients, Development, Acceptability

## Abstract

**Background:**

Inadequate energy and micronutrient intake during childhood is a major public health problem in developing countries. Ready-to-use supplementary food (RUSF) made of locally available food ingredients can improve micronutrient status and growth of children. The objective of this study was to develop RUSF using locally available food ingredients and test their acceptability.

**Methods:**

A checklist was prepared of food ingredients available and commonly consumed in Bangladesh that have the potential of being used for preparing RUSF. Linear programming was used to determine possible combinations of ingredients and micronutrient premix. To test the acceptability of the RUSF compared to *Pushti* packet (a cereal based food-supplement) in terms of amount taken by children, a clinical trial was conducted among 90 children aged 6–18 months in a slum of Dhaka city. The mothers were also asked to rate the color, flavor, mouth-feel, and overall liking of the RUSF by using a 7-point Hedonic Scale (1 = dislike extremely, 7 = like extremely).

**Results:**

Two RUSFs were developed, one based on rice-lentil and the other on chickpea. The total energy obtained from 50 g of rice-lentil, chickpea-based RUSF and *Pushti packet* were 264, 267 and 188 kcal respectively. Children were offered 50 g of RUSF and they consumed (mean ± SD) 23.8 ± 14 g rice-lentil RUSF, 28.4 ± 15 g chickpea based RUSF. *Pushti packet* was also offered 50 g but mothers were allowed to add water, and children consumed 17.1 ± 14 g. Mean feeding time for two RUSFs and *Pushti packet* was 20.9 minutes. Although the two RUSFs did not differ in the amount consumed, there was a significant difference in consumption between chickpea-based RUSF and *Pushti* packet (*p* = 0.012). Using the Hedonic Scale the two RUSFs were more liked by mothers compared to *Pushti packet*.

**Conclusions:**

Recipes of RUSF were developed using locally available food ingredients. The study results suggest that rice-lentil and chickpea-based RUSF are well accepted by children.

**Trial registration:**

ClinicalTrials.gov NCT01553877. Registered 24 January 2012.

## Background

Bangladesh has one of the highest childhood malnutrition rates in the world. The prevalence of underweight (<-2 z score weight-for-age) among children less than five years old is 36 percent and stunting (<-2 z score height-for-age), which denotes chronic malnutrition, is 41 percent [1]. Bangladesh has an estimated 600,000 children with severe acute malnutrition (SAM) and 1.8 million with moderate acute malnutrition (MAM). As a result, currently there are 2.4 million children under five years of age in the country suffering from acute malnutrition (<-2 z score weight-for-height) [1]. Malnutrition is nearly always accompanied by deficiencies of essential micronutrients, raising the importance of evaluating the impact of micronutrient content of food products in reducing micronutrient deficiencies during 6–12 months of life [2]. Although breast feeding rates have increased considerably in Bangladesh (90 percent of under-two children breast fed and 64 percent exclusively breast fed during the first 6 months of life), only 24 percent of young children are fed as per appropriate infant and young child feeding (IYCF) practices [1]. Research done in rural Bangladesh showed that complementary foods are grossly deficient in essential micronutrients [3]. In a recent study we assessed the adequacy of intake of 11 micronutrients among 24–48 months children in rural Bangladesh [4]. The overall mean prevalence of adequacy of micronutrient intakes for children was only 43 percent. The prevalence of adequacy was less than 50 percent for iron, calcium, riboflavin, folate, and vitamin B12. In the same population we observed that children consumed sub-optimal amounts of fat and in most children, only one to four percent of the total energy came from essential fatty acids [5]. These observations reflect food insecurity which affects about 20–30 percent of the population of the country, as well as low dietary diversity and low feeding frequency of young children among a larger part of the population. Although effective counseling to improve the quality of complementary feeding works in food secure communities, supplementation with nutritious food may be imperative for children, especially those who cannot afford an adequately diverse diet [6,7].

To reduce growth faltering among young children in resource constrained countries several food supplements have been developed and tested with contrasting results [8-10]. However, considering the country context, a new supplementary food made of locally available food ingredients needs to be developed in Bangladesh. This new supplementary food was designed similar to the *Pushti* packet (a mixture of roasted rice and lentil flour, with molasses and oil), the food supplement used in the erstwhile National Nutrition Program of Bangladesh. This food supplement is referred in this paper as ‘Ready-to-use supplementary food’ (RUSF) made of locally available food ingredients and designed to have the required amount of micronutrients and vitamins essential for growth and development of children 6–24 months of age. RUSF does not require cooking and can be consumed without adding water either on its own or by mixing with other food such as rice porridge. It has minimal water content and thus, the risk of contamination or bacterial growth is greatly reduced. These characteristics make provision of RUSF a safe nutrition for young children in Bangladesh. This paper aims to describe the formulation of newly developed RUSF recipes and to assess whether these RUSF are acceptable compared with the existing *Pushti* packet among children aged 6–18 months in a clinical trial design.

## Methods

### Development of RUSF

#### ***Selection of ingredients***

As part of the development of RUSF, a checklist was prepared for all food ingredients available and commonly consumed in Bangladesh that have the potential of being used for developing a RUSF. A final selection was made based on the nutritive value, local availability, and cost of the local ingredients. All ingredients were purchased from the local market. Vitamin and mineral premix was obtained from DSM Switzerland.

### Recipe formulation and production

The theoretical formulation of RUSF components was made based on linear programming to identify the combinations of ingredients that would result in the most nutritious recipes. Linear programming analysis is a powerful approach for identifying a low-cost nutritionally adequate diet [11] which is based on a mathematical iterative approach involving multiple calculations of products and sums that can be quickly performed by a personal computer [12]. The energy density of RUSF was targeted at 250 kcal/50 g (per serving), and caloric distribution was targeted to be 45–50 percent from fat and 8–10 percent from protein. Based on expert opinion and consensus within the research team, micronutrient content was set to cover 70 percent of the requirements of children aged 6–18 months. Experiments for developing recipes and preparation of samples were done at the icddr,b Food Processing Laboratory following a standardized production procedure to control the quality of RUSF from each production batch and ensure that no unexpected contamination and nutrient losses occur during processing. Potential recipes were produced in small batches by mixing all ingredients in an electric blender. When necessary, consistency of the recipe was adjusted by varying the amount of dry ingredients and soybean oil. Furthermore, the combination of minerals and vitamins were adjusted to avoid unpleasant taste which can occur with addition of high dose of micronutrients. A small amount (1 percent) of soy lecithin was added to the recipe in order to improve the consistency and prevent oil separation.

### Determination of RUSF quality and stability

Microbiological tests (total viable count, yeasts, moulds, coliforms, *Escherichia coli, Bacillus cereus, Staphylococci, Listeria monocytogenes, Cronobacter sakazaki*) were done at icddr,b Food Safety Laboratory. Chemical properties (pH, water activity, moisture, peroxide value, total aflatoxin), nutritional composition (protein, fat, energy, carbohydrates) and micronutrient composition (vitamins, and minerals) were determined at the Institute of Nutrition, Mahidol University, Thailand based on standard procedures.

In order to preliminarily assess the storage stability of RUSF, sensory quality of RUSF was assessed after two weeks of storage under ambient conditions (30.0°C, 58 percent relative humidity). Difference-from-control test was conducted by thirteen panelists from among staff of icddr,b and are caregivers familiar with feeding their children complementary food but not directly involved with the present study. Panelists received three samples (15–20 g in white plastic cups) for each formula, one stored at room temperature (test sample) and two controls (A and B) stored in a freezer. All samples were blinded to the panelist and were coded with three-digit random numbers except control sample A. They were randomly served to each panelist. Panelists were asked to rate for the degree of difference in odor and flavor of samples from the control sample A. The rating was performed on 5-point scale with 0: no difference and 4: extremely different. No difference in odor and flavor was observed between sample that was kept at room temperature and the one kept in a freezer, suggesting that the RUSF could be kept at room temperature for up to two weeks without any change in its sensory quality. There was also no change in microbiological quality of RUSF stored at room temperature over two weeks. The product development stage took nine months between January-September 2011. However, the RUSF used for the acceptability trial were freshly prepared every alternative day.

### Acceptability trial

#### ***Outcome variables***

The primary outcome variable for the acceptability trial was to see the acceptability of RUSF or *Pushti* packet by measuring the amount of food consumed by children. The secondary outcome variable was to measure children’s mothers’ opinion on the food’s color, flavor, mouth feel, and overall acceptability by using a seven point Hedonic Scale.

### Study settings

The acceptability trial was carried out in an under-privileged community living in a slum in Mirpur, Dhaka, Bangladesh. The slum in Mirpur was selected as the site of the study because it is inhabited by poor families, and represents a typical slum settlement in Bangladesh. Mirpur is one of the 27 *Thanas* of Dhaka City with a population of about one million in an area of 59 square kilometres. The acceptability study was conducted during January-February 2012. This was an open labeled study. Blinding was not done because the various types of foods were very distinct.

### Sample size

The sample size was based to test the hypothesis that the mean consumption of RUSF during the acceptability test would be at least 40 percent of the amount offered. We assumed that the standard deviation of consumption would be 15 percent of the amount offered. The sample size of 30 for each diet would therefore allow us to reject the null hypothesis with 80 percent power if the true means were at least 60 percent. The sample size was also adjusted for multiple comparisons using Bonferonni correction.

### Enrollment

All children in the community aged 6–18 months were screened for nutritional status and presence of any illness. Upon fulfilling the enrolment criteria (age 6–18 months, started semi-solid food) and receiving the consent for participation in the study from the parents or legal guardians, the children together with their respective mother/caregiver were randomly allocated into three different study groups and children were enrolled. Children did not meet the enrolment criteria if their weight-for-age or weight-for-height z-score was < -3, if they had any acute illness or features suggestive of any chronic disease such as tuberculosis, any congenital anomalies such as trisomy 21, cleft lip or palate.

### Randomization

**Figure 1:**
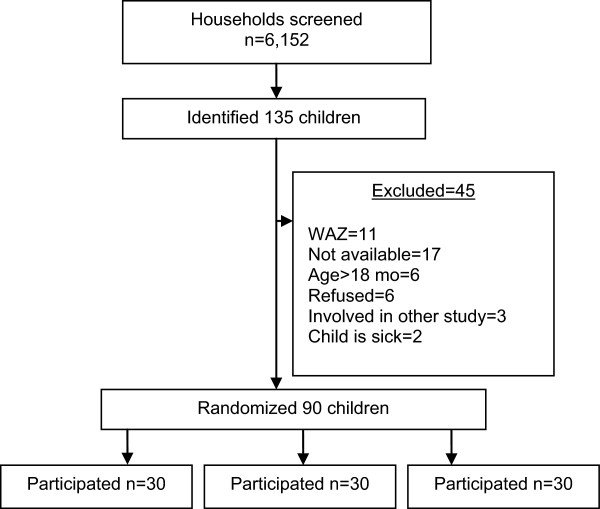
A trial profile.

A total of 135 children from 6,152 households were identified (Figure 1) for randomization. Of these children, 90 children were assigned to three different study groups (rice-lentil RUSF, chickpea based RUSF or *Pushti* packet groups) using simple random sampling according to computer-generated random numbers. Computer generated numbers were given by another researcher within the same organization but not involved with the existing study.

### Intervention

*Pushti* packet was offered at 50 g. In order to maintain comparability, each RUSF was also offered at 50 g daily. However, the total energy obtained from *Pushti* packet was not equal to RUSF. The energy and nutrient content of the three foods i.e. rice-lentil-based RUSF, chickpea-based RUSF and *Pushti* packet is shown in Table 1. *Pushti* packet was prepared using roasted rice powder (26.3 g), roasted lentil powder (13.2 g), molasses (6.6 g), and soybean oil (3.9 g) per serving. It does not contain any added micronutrients. Therefore, in addition to existing ingredients of *Pushti* packet, we gave one sachet of *Pushtikona* (Renata Limited, Dhaka)*,* which is a micronutrient powder containing 15 micronutrients (vitamin A 0.40 mg, vitamin C 30 mg, vitamin D 0.005 mg, vitamin E 5 mg, Thiamine 0.5 mg, riboflavin 0.5 mg, niacin 6 mg, pyridoxine 0.5 mg, Cyanocobalamin 0.0009 mg, folic acid 0.15 mg, iron 10 mg, zinc 4.1 mg, copper 0.56 mg, selenium 0.017 mg, iodine 0.09 mg). While giving *Pushti* packet we allowed mothers to add water to the food mix as per requirement, and asked them to add the *Pushtikona*.

**Table 1:** **Composition of RUSF and *Pushti *** **Packet per 50 g (per serving)**

	**Rice-Lentil based RUSF**	**Chickpea based RUSF**	***Pushti *** **packet**
Energy (Kcal)	264	267	188
Moisture (g)	1.0	1.2	ND
Protein (g)	5.1	6	4.9
Total fat (g)	14.8	15.9	4.2
Carbohydrate (g)	27.6	24.9	32.6
Dietary fibre (g)	1.1	0.6	ND
Ash (g)	1.95	2.5	ND
Vitamin A (μg)	427.5	294	0.39
Β carotene (μg)	8.5	26.5	ND
Vitamin C (mg)	16	20.5	0.65
Vitamin E (mg)	10.41	14.0	ND
Vitamin B_1_ (mg)	0.475	0.78	0.07
Vitamin B_2_ (mg)	0.515	0.63	0.04
Calcium (mg)	286.0	413.3	0
Phosphorus (mg)	240	318	ND
Sodium (mg)	20.5	37	ND
Potassium (mg)	317	424.5	ND
Magnesium (mg)	51.425	71.5	ND
Iron (mg)	5.905	7.0	1.92
Copper (mg)	0.34	0.4	ND
Zinc (mg)	4.15	4.9	0.80
Chloride (mg)	38.5	68.5	ND
Aflatoxin	Not detected	Not detected	ND
Water activity (24.6˚C)	0.32	0.32	ND
pH	6.3	4.1	ND
Peroxide value	0.2	0	ND

ND: Not determined.

### Observation of feeds and interviews with caregivers

The feeding on first day was held at the nutrition centre in Baoniabad slum of Mirpur, Dhaka. The first day feeding session enabled study staff to get familiarized with the mothers and children and also for the latter to be habituated to the food. After the first day feeding session all participants were supplied with the respective food supplement for two days to use under real life conditions with a daily dose of 50 g. Before end of first two days supplementation, our field worker visited the households and continued her visit in the households every alternate day to give the supplements and recorded morbidity, if there was any. At the end of one week period, field workers requested the participants to come again to the nutrition centre and the feeding was observed for the second time. Data on this second day at the end of the one week period was included in the analysis.

The RUSF was prepared each morning by our health workers in icddr,b Food Processing Laboratory under supervision of the investigators, and then carried to the nutrition centre in Mirpur. On the first day of the study, information was sought on the families’ wealth, standard of housing, family structure and parental characteristics. A trained research assistant recorded the children’s nude weight or with light clothing using a digital scale with 10 g precision (Seca, model-345), length (using a calibrated length board), and mid upper-arm circumference to the nearest mm (using a non-stretch insertion tape). We ensured that infants were offered the assigned diet at least 2 hours after they were last fed.

During the feeding time, the mothers were asked to spoon feed their children the assigned diet until the child refused to eat. After a two-minute pause, the same food was offered a second time until s/he refused again. After a second two-minute pause, the food was offered a third time until refused again. After this third refusal, the feeding episode was considered terminated. The duration of the feeding (excluding the intervening ‘pause periods’) was recorded by stopwatch, and the total duration of the feeding was noted. The feeding episode took place under the direct supervision of a trained research assistant to make sure that feeding was not forceful. Children were considered as refusing intake if they moved their head away from the food, cried, clamped the mouth shut or clenched the teeth, or became agitated, spit out the food or refused to swallow. The amount of food ingested was calculated by subtracting the left-over from the offered amount. Pre-weighed napkins were provided; any food that was regurgitated, vomited or spilled was swabbed, the napkin weighed and subtracted from the weight of the amount offered. Using a 7-point Hedonic Scale in which each point (1 = dislike extremely, 2 = dislike moderately, 3 = dislike, 4 = neither dislike nor like, 5 = like slightly, 6 = like moderately, 7 = like extremely) was depicted by a facial drawing, we asked mothers to rate the food’s color, flavor, mouth feel, and overall acceptability.

### Analysis

We performed data analysis using SPSS version 16. Background characteristics of the participants were evaluated by using descriptive statistics. For the acceptability test, we calculated the percent of RUSF that children consumed as well as mean ± SD of the amount of the RUSF. We used one-way ANOVA and post-hoc Bonferonni test to detect differences in continuous variables, and chi-squared tests for proportions. Data from the Hedonic Scale questions were presented as mean ± SD.

### Ethical approval

Ethical approval was obtained from icddr,b Institutional Review Board. Informed and signed consent were obtained individually from the caregivers of the participants in the study, and all data were coded to remove identifying information and secure confidentiality. The trial was registered at Clinical trials.gov and the registration number of this trial is NCT01553877.

## Results

### Development of RUSF

**Figure 2:**
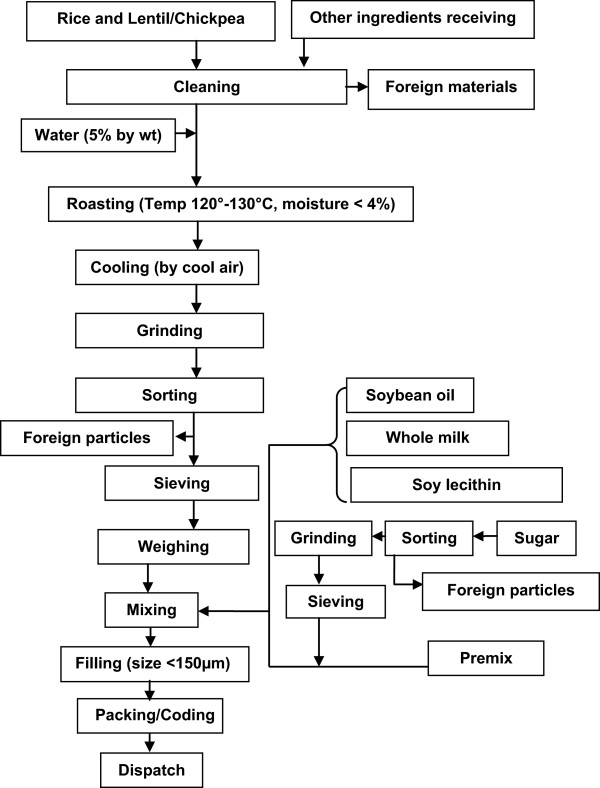
Flow diagram of RUSF production.

Rice, lentil and chickpea were chosen as ingredients for making RUSF. These ingredients are widely grown and consumed in Bangladesh and other South Asian countries. Two varieties of RUSF were developed - one was rice and lentil based and the other was chickpea based. Dried skimmed milk powder, sugar, soybean oil and vitamin mineral premix were the common ingredients for both RUSF. The total energy content of 50 g of rice-lentil and of chickpea-based RUSF was 264 kcal and 267 kcal respectively. Protein-energy ratio (PER) for rice-lentil and chickpea recipes were 7.7 and 8.9 percent respectively, whereas fat-energy ratio (FER) for the two recipes were 50.4 percent and 53.6 percent respectively. These RUSF had greater energy density than *Pushti* packet (energy 188 kcal per 50 g, PER 10.4 percent, and FER 20.1 percent). Preparation of RUSF undergoes different steps i.e. roasting, particle size reduction, homogeneous blending and packaging (Figure 2).

### Acceptability trial

A total of 135 children were identified in the community of whom 90 children were found eligible; they were enrolled and completed the trial. They included 52 girls (57.8 percent) and 38 boys (42.2 percent), and their mean age was 13.9 ± 2.9 months. Mean years of household head’s education was 6.0 ± 3.8 years (Table 2). Individual or household characteristics did not differ significantly by study groups (Table 2).

**Table 2:** Characteristics of children who completed the study

**Characteristics**	**Rice-Lentil based RUSF (n = 30)**	**Chickpea based RUSF (n = 30)**	***Pushti *** **packet (n = 30)**
	**(1)**	**(2)**	**(3)**
Child characteristics			
Age (mo), mean ± SD	14.1 ± 2.9	13.9 ± 2.6	13.8 ± 3.2
Sex% Girl (n)	60 (18)	53.3 (16)	60 (18)
Weight, Kg (mean ± SD)	8.2 ± 0.6	8.3 ± 0.7	8.1 ± 0.7
Height, cm (mean ± SD)	72.7 ± 3.5	73.2 ± 3.2	71.5 ± 4.4
MUAC, mm (mean ± SD)	139.1 ± 7.2	137.7 ± 7.4	136.6 ± 6.8
Weight-for-height z score < -2,% (n)	16.7 (5)	3.3 (1)	6.7 (2)
Height-for-age z score < -2,% (n)	36.7 (11)	36.7 (11)	50.0 (15)
Weight-for-age z score < -2,% (n)	33.3 (10)	26.7 (8)	36.7 (11)
Household characteristics			
Family size (mean ± SD)	4.43 ± 1.3	4.9 ± 1.9	4.6 ± 2.0
Household head completed primary education,% (n)	73.3 (22)	60.0 (18)	63.3 (19)
Using shared latrine,% (n)	80.0 (24)	86.3 (26)	80.0 (24)
Tap water source of drinking water,%	96.7 (29)	96.7 (29)	93.3 (28)

Children consumed on an average 23.1 ± 15.4 g of offered food which took them 20.9 ± 9.6 mins (Table 3). Amounts of food consumed by rice-lentil and chickpea-based food groups, and the times taken to consume them did not differ significantly. Children consumed an average of 47.1-56.7 percent of the RUSF and 34.4 percent of *Pushti* packet offered. There was a significant difference (*p* = 0.012) in amount of chickpea-based RUSF and *Pushti* packet consumed (the former was better) (Table 3). On the 7-point Hedonic Scale, mean response for each sensory quality (color, flavor, mouth feel, and overall liking by mother’s opinion) of all foods was more than 6. Rice-lentil, and chickpea-based RUSF were significantly better compared to *Pushti* packet in terms of ‘overall liking’ (Table 3).

**Table 3:** Results of test feeding of RUSF

	**Rice-Lentil based RUSF (n = 30)**	**Chickpea based RUSF (n = 30)**	**Pushti packet (n = 30)**	***P *** **value**^**1**^	***P *** **value**^**2**^		
	**(1)**	**(2)**	**(3)**		**1 *vs*.2**	**2 *vs.*** **3**	**1 *vs.*** **3**
Mean Hedonic Scale (mean ± SD)							
Color of the supplement	6.9 ± 0.2	6.9 ± 0.3	6.8 ± 0.4	ns	-	-	-
Aroma/flavor of the supplement	6.8 ± 0.4	6.8 ± 0.3	6.5 ± 0.6	0.042	ns	0.049	ns
Texture/mouth feel	6.7 ± 0.4	6.8 ± 0.3	6.2 ± 0.7	0.000	ns	0.000	0.002
Overall Liking	6.9 ± 0.2	6.9 ± 0.3	6.4 ± 0.6	0.000	ns	0.000	0.000
Amount offered, g (mean ± SD)	50.4 ± 0.5	50.2 ± 0.5	49.9 ± 1.2 (118.0 ± 12*)	ns	-	-	-
Amount consumed, g (mean ± SD)	23.8 ± 14	28.4 ± 15	17.1 ± 14 (40.8 ± 35*)	0.015	ns	0.012	ns
Percent of food consumed from offered food (%)	47.2 ± 28	56.7 ± 31	34.4 ± 28	0.017	ns	0.014	ns
Energy received from consumed food (mean ± SD), Kcal	125 ± 76	152 ± 83	64 ± 53	0.000	ns	0.000	0.005
Feeding time, minutes (mean ± SD)	23.0 ± 10	20.7 ± 10	20.4 ± 7	ns	-	-	-
Velocity, g/min	1.3 ± 1.0	1.9 ± 2.2	0.8 ± 0.8	0.020	ns	0.016	ns

ns: Not significant at 5% level; *after adding water; ^1^ANOVA; ^2^post-hoc.

Interviews with the caregivers/mothers with the structured questionnaire revealed that 18/30 children (60%) liked rice-lentil and 20/30 children (66%) preferred the chickpea-based RUSF. In *Pushti* packet group, only 12/30 (40%) caregivers reported that their children liked the supplement. The common reason stated by the mother for her child’s liking was that the child ate most of the portion served. Almost one third of the caregivers felt that the consistency of the RUSF was appropriate for children. Fifteen caregivers felt that the consistency of rice-lentil (5/30) and chickpea-based (10/30) supplements was thick. Some caregivers reported that rice-lentil (10/30) and chickpea based (9/30) supplements were too sweet in taste, whereas *Pushti* packet study participants (11/30) reported the taste was neither sweet nor salty. Few mothers (5/60) in rice-lentil and chickpea based RUSF study groups mentioned that the food had a strong taste which is more like a medicine.

## Discussion

Our results suggest that rice-lentil and chickpea-based RUSF were more acceptable than *Pushti* packet, which was the least acceptable of the three foods studied. The assessment of the acceptability of the three food supplements was a bit challenging because we had to depend partially on the opinion of mothers whose tastes and food preferences, as adults, are different from those of the children. Although our primary objective was to measure the mean proportion of offered food consumed by the children but we also measured the mean Hedonic Scale score. Our rationale was that with no forced feeding, the amount of the offered food consumed by children would depend largely on the extent to which they liked the food, given that none of the children were fed for at least 2 hours prior to the feeding session. Children consumed an average of 47.1-56.7 percent of the RUSF offered and 34.4 percent of *Pushti* packet offered. Mean Hedonic Scale score for *Pushti* packet was also significantly lower compared to the two RUSF. We can, therefore, say with reasonable certainty that children accepted rice-lentil and chickpea based RUSF more compared to *Pushti* packet. The Hedonic Scale responses from the mothers suggested a high level of acceptability, but as observed in a similar study in Mexico and Ghana [8,13], such Hedonic Scale responses may not be conclusive because respondents could be reluctant to give any negative comment [14]. Our mean Hedonic Scale across all three foods was high suggesting this may have also occurred in our study. On the other hand, total consumption of the offered food is an option to assess the acceptability of food supplements [8,10]. Thus, combining the results from Hedonic Scale testing and total consumption is probably the best way to judge the acceptability of the RUSF.

The concerns raised by few caregivers about the taste of RUSF being similar to a medicine warrants discussion. Our general observation was that caregivers considered the supplement as food, and therefore, they seem to expect it to taste much the same way as a typical food might taste. The relatively high mineral concentration makes any attempt to get the supplement to taste like a typical food a challenge, from a food technology perspective [3]. There may be a way to deal with the medicinal taste issue: it should be explained to caregivers that the RUSF, although it looks like food, has a high nutrient concentration and therefore may not taste like a normal food to few caregivers. Indeed it would be a point for counseling when the product will be launched at larger scale in a nutrition program.

*Pushti* packet was found less acceptable among children and caregivers. This supplement had one major characteristic that contributed to its lesser acceptance by the children or caregivers, which is: adding water to *Pushti* packet increases the volume of food and in turn it reduces the sweetness. Moreover, the fat content is less than RUSF. This study has provided useful insights for the efficacy trial of RUSF which is now being conducted in Gaibandah, in the northern part of Bangladesh, in collaboration with John Hopkins University and the World Food Programme. In this efficacy trial, approximately 5000 children, 6–18 months old, have been randomly assigned to the two locally produced RUSF, Supercereal Plus (also known as wheat soya blend plus plus or WSB++) and a commercially available, imported food supplement called Pumpy’Doz™. The trial will not only evaluate the effects of RUSF on the nutritional status of children, but will also provide extensive information on the long term acceptability of these products and add to what we have learned from this acceptability study.

## Conclusion

We developed two RUSF based on locally available food ingredients. We conclude that the newly developed rice-lentil and chickpea-based RUSF are acceptable to children and their caregivers. This study presenting acceptability data on locally produced RUSF for children, which is a novel way to ensure nutritional adequacy of children’s diet particularly those living in food insecure conditions, and is nutritionally more complete than *Pushti* packet.

## Competing interests

Authors do not have any conflict of interest in writing this paper.

## Authors’ contribution

Conceptualized the work, participated in data collection and management, and drafting of manuscript: TA, NC, MIH, NT and MMI. Provided technical assistance and contributed to the manuscript: GS. Helped in interpretation of findings, and contributed to the critical revision of the manuscript for making the final draft for submission: SdP, GS, RF, SAS, MP, KPW, and PC. All authors approved the draft.

## Pre-publication history

The pre-publication history for this paper can be accessed here:

http://www.biomedcentral.com/1471-2431/14/164/prepub
